# Socio-Demographic and Attitudinal Correlates of Problematic Social Media Use: Analysis of Ithra's 30-Nation Digital Wellbeing Survey

**DOI:** 10.3389/fpsyt.2022.850297

**Published:** 2022-02-28

**Authors:** Justin Thomas, Marina Verlinden, Fahad Al Beyahi, Bahiah Al Bassam, Yasmin Aljedawi

**Affiliations:** ^1^Department of Psychology, College of Natural and Health Sciences, Zayed University, Abu Dhabi, United Arab Emirates; ^2^King Abdulaziz Center for World Culture, Dhahran, Saudi Arabia

**Keywords:** social media use, social media disorder, internet addiction, international, national culture

## Abstract

Time spent on social media continues to rise globally. For some individuals, social media use can become maladaptive and associated with clinically significant social and occupational impairments. This problematic social media use (PSMU) is also linked with poorer health and wellbeing. Much of our existing PSMU knowledge comes from single nation studies, heavily focused on adolescent and college-age samples. This study uses data from Ithra's 2021 global digital wellbeing survey to explore rates of PSMU and identify socio-demographic and attitudinal correlates. Participants (*N* = 15,000) were representative adult samples (*N* = 500) drawn from 30 nations. All participants provided socio-demographic data and completed a measure of PSMU, along with questions assessing attitudes toward social media and general usage patterns. PSMU prevalence was 6.82%, varying from 1.7 to 18.4% between nations. Multivariate logistical regression identified several correlates, including national culture, age, parenthood and frequency of use. These findings can help inform public policy and public health initiatives to reduce PSMU prevalence.

*“…new technology does not add or subtract, it changes everything. In the year 1500, 50 years after the printing press was invented, we did not have the old Europe plus the printing press—we had a different Europe”* (1).

## Introduction

One of the most widespread and rapidly embraced information age technologies is social media. In the 25 years since the first primitive platforms emerged, the number of active users has grown exponentially, reaching around 4.48 billion in July 2021 (2). While the definition of “active use” varies, it generally denotes individuals who log in and perform actions that facilitate direct exchanges with other users, for example, sharing messages, posting images, and commenting on peer-content (3). Social media use among US adolescents increased markedly between 2010 and 2015 (4). International industry data extend and confirm this trend, with social media users averaging 145 min per day in 2020, up from 90 min per day in 2012 (5).

Beyond the usage norms, however, there are also concerns that social media use can become maladaptive, resembling a behavioral addiction or an impulse control disorder (6–10). This evolving concept of a pathological form of social media use is typically traced to the work of Griffiths (11, 12). Building on the work of Brown (13), Griffiths proposed a “components” model of addiction, arguing for a common set of symptoms (salience, mood modification, tolerance, withdrawal, conflict and relapse) shared by all addictions, including behavioral addictions. For Griffiths, this could be extended to video games (14), internet use (15) and more. Further pioneering work in online addictions drew on the DSM-IV (16) conceptualization of pathological gambling (9). Young proposed a set of diagnostic criteria for internet addiction disorder (preoccupation, tolerance, withdrawal, relapse/persistence, mood modification/escape, deception, displacement, and conflict) that have subsequently influenced the conceptualization and measurement of gaming (17) and social media (18) disorder (19). Young's internet addiction concept is broad, comprising five subtypes, including cybersexual addiction, gaming addiction, internet compulsions (online gambling/shopping), compulsive information seeking, and cyberrelationship addiction (10). This last subtype–cyberrelationship addiction-comes closest to our contemporary ideas about maladaptive social media use.

The current nomenclature attached to compulsive and dysfunctional social media use includes social media disorder, social media addiction, or compulsive social media use. However, perhaps due to the lack of conceptual clarity and consensus, the term “problematic social media use” (PSMU) has also gained traction in recent years. For example, between July 2020 and July 2021, PubMed included 26 articles with the phrase “problematic social media use” in the title; for “social media addiction”, there were 17, and for “social media disorder”, just 4. Despite these concerns over social media use, contemporary psychiatric nosology in the form of the ICD-11 (20) and DSM-5 (21) have yet to include social media addiction/PSMU as an official diagnostic entity. Gaming disorder was included in the ICD-11 in 2019 as a behavioral addiction.

Because currently PSMU is not listed in DSM-5 or in ICD-11 as a preliminary or formal diagnostic entity, there is some controversy in conceptualization and in distinguishing between normal and maladaptive use of social media. On one hand, PSMU can be seen as part of Internet addictions in general. For example, aligned with the DSM-IV (16) criteria for pathological gambling, Young (9) proposed a set of diagnostic criteria for Internet addiction disorder, variously listed as: preoccupation, tolerance, withdrawal, persistence, mood modification, deception, displacement, and conflict. Young's et al. (10) concept of Internet addiction is overarching, comprising at least five subtypes: cybersexual addiction, gaming addiction, Internet compulsions (online gambling/shopping), compulsive information seeking, and cyberrelationship (social media) addiction. From this perspective, PSMU can be seen as part of Internet addiction and may well co-occur with other its known subtypes. And while some of the subtypes of the Internet addiction are becoming recognized as a potential independent diagnosis, to date, however, there remains a lack of conceptual clarity and nosological consensus concerning maladaptive social media use. Several definitions of problematic social media use can be found in the literature. For example, social network site addiction is a commonly used term in research in this field (22, 23), and it emphasizes the same aspects of addiction that are seen in substance and behavioral addictions, namely salience, tolerance, mood modification, conflict, withdrawal, problems, and relapse (23). Some researchers have taken on an approach of applying the diagnostic criteria of the Internet gaming disorder to the maladaptive and compulsive use of social media and referred to it as social media disorder (18). Other researchers (24) use a term problematic social networking site use (PSNSU), while pointing out that terms as diverse as: Facebook addiction, addictive use of social networking sites, Internet-communication disorder, smartphone use disorder, smartphone addiction, and Internet communication/social networks use disorder have also been used in the field (24). The latter term (Internet communication/social networks use) was suggested to be used as a part of Internet use disorders distinguishing between mobile and non-mobile Internet use disorders (25), however this taxonomy remains work in progress as the field still requires clarity on what criteria can be used to classify an online behavior as disorder besides the over-use as such (24).

Considering the controversies with conceptualizing the maladaptive use of social media, and considering it is not currently outlined as a formal disorder, we follow the common approach in this field of research when referring to this maladaptive online behavior, and use a broader term in our study, namely-problematic social media use (PSMU). And while no common definition of PSMU exists to date, it is commonly interpreted as a maladaptive form of social media use that is characterized by loss of control, compulsiveness and experiencing negative consequences from the excessive use of social media such as negative impact on daily life, performance at work or school, social activities, interpersonal relations and on the overall health and sense of wellbeing (22, 26). Problematic social media use, however, remains a condition for further study.

Much of this further study has investigated the prevalence of PSMU, associated morbidities and its implications for health and wellbeing. There are now several meta-analytic reviews reporting epidemiological data for PSMU. Notably, Cheng et al. (27) combined data from 62 independent studies, including 34,798 participants from 32 nations. The pooled prevalence for PSMU in their analysis was 5%. This figure was arrived at using the most stringent screening criteria, counting only those individuals classified as experiencing “very severe symptoms”. When relaxed criteria were used, the pooled prevalence rose to 13%, with a high degree of international variability. Cheng et al. also grouped nations by national cultural values, specifically, the degree to which each society emphasized individualist (independence) vs. collectivist (interdependence) values (28). Rates of PSMU were significantly higher among collectivist (e.g., Japan, India, Taiwan) populations (31%) compared to their individualist (e.g., USA, UK, Australia) counterparts (14%). The influence of cultural values on PSMU remains an area for future research (27).

Another meta-analysis explored gender-related differences in PSMU and gaming disorder (29). This analysis included 53 independent studies, including 82,440 participants from 21 nations/regions. Across the whole sample, there were relatively higher levels of PSMU among women (small effect size), while men were more likely to meet the screening criteria for gaming disorder (moderate effect size). Again, however, these findings demonstrated significant international variability. For example, India's pattern showed larger gaming disorder effects in females and PSMU in males (29). Similarly, for PSMU, gender-related differences in effect sizes were larger in Asian nations than their European and North American counterparts. These findings, again, highlight the need for studies to get beyond single nation data and explore PSMU internationally and cross-culturally.

Beyond meta-analytic studies, extensive multinational primary research studies of PSMU are scarce. However, Boer et al. (30) explored data from the 2017/2018 Health Behaviors in School-aged Children (HBSC) survey. This multinational survey assessed PSMU and wellbeing (life satisfaction, school satisfaction, family and peer-support) among 154, 981 adolescents across 29, primarily, European countries (30). The average PSMU prevalence across territories was 7.38%, ranging from 14.17% for Spain to a low of 3.22% for the Netherlands. PSMU was, without exception, negatively associated with poorer scores across all the wellbeing domains. This study also looked at “intense social media use”, defined as high-frequency use, without behavioral addiction symptomatology. Across all nations, 34.03% of adolescents were categorized as intense users. Unlike PSMU, however, intense use was not unequivocally associated with poorer wellbeing. In those nations where intense social media use was a norm (high prevalence of intense users), it was even associated with higher levels of wellbeing. This study highlights the critical distinction between frequent/intense and problematic use, where frequent use is not necessarily problematic and may even be associated with better wellbeing in specific contexts. Rather than frequency/intensity of use, features such as loss of control and preoccupation are more likely to underpin PSMU's observed association with poorer wellbeing (30).

While shedding important light on PSMU, these studies have several limitations. First, the individual studies included in the meta-analyses were undertaken in different years, at different times of the year. These temporal differences may impact findings, especially when attempting to compare nations, regions, or cultures. Furthermore, the current conceptual ambiguity surrounding PSMU means that the pooled studies frequently rely on different screening tools to identify cases. This may introduce variations due to methods used. Furthermore, most of the individual studies in the pooled meta-analyses focus on young people (school and college-age students), disproportionately from Europe and North America (29). This Western focus is also true of the one large multinational study we were able to identify.

The present study aims to address some of these previous limitations. Using multivariate logistic regression, it also aims to identify and clearly communicate the socio-demographic and attitudinal correlates of PSMU across a diverse multinational sample of adults aged 18 to 91. In addition, our study examines PSMU data from a multi-regional (Americas, Middle East, Africa, Asia) digital wellbeing survey administered to a sample of 15,000 adults across 30 territories within a 1-month data collection window. These multinational data provide an opportunity to identify and describe regional differences while exploring socio-demographic and attitudinal variables associated with PSMU. Based on previous research, we formulated the following research hypotheses about the PSMU and age of the participants as well as regarding the PSMU and national culture. Our first hypothesis is in line with Cheng et al. (27) where we hypothesize that, after adjusting for other socio-demographic variables, collectivist nations will report higher rates of PSMU than their individualist counterparts. Similarly, in line with earlier research (31), we predict higher rates of PSMU among participants from the younger age group. However, we make no specific directional predictions regarding PSMU's association with the study's other variables.

## Methods

### Participants

The 30-nation digital wellbeing survey (DWS) was commissioned by King Abdulaziz Center for World Culture (Ithra). Data collection was managed by PSB Insights, a global analytics consultancy with a 40-year track record in offering research and polling services across more than 100 countries. PBS routinely conduct extensive multinational surveys, a notable example being the annual Arab Youth Survey, currently in its 13th year. For the DWS, all materials were translated and back-translated from English into the majority language of each of the 30 participating territories. The samples in each nation were probability-based representative draws of 500 individuals. Participants were drawn at random from the pre-existing panels. Depending on the nation, panel sizes varied from 10s of thousands to millions. In each country, a representative sample of the adult population (18 years old and older) was ensured by applying demographic quotas (stratification), including age, gender, region, and nationality (in some countries). Potential participants were excluded if they were under 18 or not literate in the major language of the territory they reside in. Thus, the final survey was administered online to nationally representative samples of 500 internet-using adults within each participating territory. Respondents were drawn from existing participant banks (panels) with participation incentives offered following local practices. The final anonymized sample totaled 15,000 individuals, with all data collected between June 12th and July 11th, 2021. Ithra's internal review board granted the ethical approval for the study (IRS 202171). Because the samples from each nation were drawn using probability-based random sampling method, and they were drawn from the representative existing panels of the data collection agency, it allows for the generalization of the findings to larger populations from which the national samples were drawn.

### Sample Characteristics

The median age of participants across the whole sample was 36 years (age range 27–50 years), 10,618 (70.8%) reported using social media. Social media users were slightly younger, Mdn = 35 (age range 27–48 years), than their non-using counterparts. Table 1 above displays additional sample characteristics.

**Table 1:** Sample characteristics: frequency counts and percentages for the study's main demographic variables.

**Variable**	**Frequency (%) Whole sample**	**Frequency (%) Social media user**
Gender		
Female	7,201 (48.0%)	5,254 (49.5%)
Male	7,799 (52.0%)	5,364 (50.5%)
Age group		
Over 35 yrs.	7,662 (51.1%)	5,227 (49.2%)
35 yrs. and under	7,338 (49.9%)	5,391 (50.8%)
Completed College		
No	6,950 (46.3%)	4,731 (44.6%)
Yes	8,050 (53.7%)	5,887 (55.4%)
National culture		
Individualist	4,500 (30.0%)	3,080 (29.0%)
Collectivist	10,500 (70.0%)	7,538 (70.9%)
Household income		
High (above national median)	6,918 (46.1%)	4,993 (47.0%)
Low (national median or less)	8,082 (53.9%)	5,625 (53.0%)
Number of children under 18 yrs.		
None	7,757 (51.7%)	5,230 (49.3%)
One	3,374 (22.5%)	2,543 (23.9%)
Two	2,555 (17.0%)	1,940 (18.3%)
Three-Five	1,207 (8.0%)	849 (8.0%)
More than 5	107 (0.7%)	56 (0.5%)

### Measures

The DWS is an extensive battery, including over 300 items spanning a broad range of demographics and digital wellbeing related topics. However, the present analysis focuses specifically on items related to social media use.

#### Problematic Social Media Use

The DWS contained eight items explicitly assessing problematic social media use. This content was based on simplifying items from the Bergen Social Media Addiction Scale (32) and the Social Media Disorder Scale (18). This simplification was deemed essential to facilitate content translation across multiple different languages. In line with Young's (9) work on internet addiction, the unequivocal endorsement of at least five items/symptoms (“strongly agree”) was used as a cut-off for the assignment of problematic use status. This cut-off is also aligned with existing DSM-5 criteria for diagnosing pathological gambling (33). The eight items included on the DWS are listed in Table 2 in descending endorsement order.

**Table 2:** PSMU Items listed in descending order of endorsement.

**Item**	**Symptom**	***M***	***SD***
I normally use social media for longer than I initially intended	Persistence	2.72	1.030
I sometimes find it difficult to disconnect from social media	Persistence	2.46	1.059
I spend less time than I should doing other day to day activities (such as household chores), because of the time I spend on social media	Displacement	2.39	1.041
I have fewer other interests (such as hobbies and other entertainment activities) because of using social media	Displacement	2.38	1.044
I need to spend increasing amounts of time engaged on social media to enjoy it	Tolerance	2.22	1.010
I feel irritability, anxiety or sadness when I stop using social media or can't use it	Withdrawal	2.05	1.029
I have misled friends, family members, or others about the amount of time I spend using social media.	Deception	1.94	1.016
Using social media has caused problems between me and my family/friends.	Conflict	1.93	1.001

*The internal reliability for the PSMU measure in the present study was good, Chronbach's α = 0.878*.

#### Demographic Items

Individual-level demographic items included nationality (30 nations), gender, age, education level, number of children under 18 years of age, household income. Except for age, responses to these demographic items were nominal or ordinal data. For the present analysis, however, responses to most variables were dichotomized. This dichotomization gave us high (above the national median) vs. low household income (median or below), children vs. no children, completed college vs. never completed college and two age groups based on a median split: over 35 vs. 35 and under. Additionally, we included national culture data for each participant based on the Hofstede Insights scores for each nation (28). Specifically, we included scores on the individualism dimension (0 to 100), which allowed us to categorize participants as belonging to either an individualist (individualism score > 50) or a collectivist society. Based on Hofstede's quantitative model of national culture (28), *individualist* societies are generally characterized by their emphasis on the “personal”. In individualist societies, personal freedoms, and individual achievements are particularly important. People are expected to be independent, to take care of themselves and their immediate family, making their own choices. The opposite end of this dimension is referred to as *collectivism*. In contradistinction to individualist societies, collectivist societies are viewed as emphasizing group harmony over and above individual achievements. Interdependence rather than independence is valued, and the cohesion and wellbeing of the broader social group is given precedence over and above personal interest and individual gain. Some hallmarks of collectivist societies within this framework include living in larger extended families, and higher rates of consanguineous (cousin) marriage (34). The dichotomization/categorization of continuous variables is commonly used in health research to stratify participants according to putative risk/resilience factors, thereby facilitating the development of easily communicated models of greatest need/highest risk (35). However, for bivariate correlational analyses, we also retained and used the continuous values for individualism (scores range 13 to 91) and age (age range 18 to 91 years).

#### Attitudinal and Behavioral Items

In addition to demographics, the survey also captured information about self-reported social media use and attitudes toward this technology. The survey asked about daily usage patterns: time spent (per day) on social media. The response options ranged from “<10 min per day” to “4 h or more per day”. The survey also asked participants what impact social media had on their quality of life. Quality of life was not assessed as a separate variable. The respondents were asked to report whether they felt that social media impacted their quality of life by either improving or reducing it. The response options to this item were: “it generally improves my quality of life” or “it generally reduces my quality of life”. Finally, participants were also asked if they had ever successfully undertaken an intentional period of abstinence (detox) from social media. For the present analysis, all responses to these variables were again dichotomized. This categorization gave us intense/frequent users (2 h or more per day) vs. infrequent users (<2 h per day), those who felt social media improved their quality of life (QoL) vs. reduced their QoL, and those who had and had not successfully abstained from social media for at least 1 week.

#### Analysis

Descriptive and inferential analyses were conducted to examine the hypotheses and to reach the objectives of the study. First, means along with the standard deviations, and where appropriate, medians with interquartile range were calculated for all the continuous variables. The proportion scores were calculated for the categorical variables. Independent sample *T*-test along with the Cohen's d effect size estimates were used to examine the group differences (gender, national culture) for PSMU scores. Inferential analyses allowed us to examine the relationships between potential predictors and PSMU. Pearson's bivariate correlation coefficients were calculated to test the associations between continuous variables. Bivariate and multivariate logistic regressions were conducted to examine the predictors of PSMU across individual sets of associations followed by adjusted analyses in which the associations were adjusted for covariates. Bivariate and multivariate logistic regressions were conducted with R (R Core Team, 2020), using generalized linear models in the base package.

## Results

### Descriptive Analysis

PSMU scores were normally distributed (*M* = 17.98, *SD* = 6.00), while age was slightly left-skewed due to a larger number of relatively young people in the sample (*Mdn* = 35.0, IQR = 21.0). The mean number of PSMU symptoms strongly endorsed was 1.16 (*SD* = 1.75). The percentage of participants scoring above the PSMU cut-off (strong endorsement of 5 or more of the eight symptoms) was 6.82%. Mean PSMU scores by nation are detailed in Table 3. A further breakdown of PSMU percentages by socio-demographic and attitudinal variables are detailed in Table 4.

**Table 3:** Mean PSMU by nation in descending order of percentage above the cut-off.

**Nation**	***N***	***M***	***SD***	***% PSMU***
India	369	21.631	6.406	18.4
Pakistan	330	20.555	5.580	13.6
Indonesia	341	19.023	5.686	11.7
Ghana	416	19.762	5.462	10.8
Kuwait	320	20.153	5.150	10
UAE	323	20.087	5.226	9.9
Egypt	380	21.045	4.553	9.7
South Africa	423	18.478	5.900	9.2
Kenya	435	19.32	5.335	9.2
Mexico	399	17.278	6.121	8.5
Saudi Arabia	316	20.737	4.955	7.9
Nigeria	430	19.193	5.127	7.7
Algeria	329	19.283	5.159	7.3
Turkey	385	19.906	4.769	7.3
Brazil	391	17.829	5.548	6.4
China	341	21.150	4.886	6.2
Germany	287	14.387	6.594	5.6
France	310	15.168	6.100	5.5
US	330	15.476	6.615	4.7
Colombia	376	16.689	5.387	4
Russia	360	17.211	5.735	3.9
Japan	255	16.898	5.389	3.9
Singapore	364	19.184	5.189	3.3
Sweden	346	14.269	5.796	3.2
Argentina	385	15.956	5.312	3.1
Australia	355	15.397	5.981	3.1
Canada	327	14.945	5.869	2.4
Italy	351	14.752	5.681	2.3
UK	351	14.761	5.705	2
South Korea	293	17.038	5.643	1.7

**Table 4:** Bivariate and multivariate logistic regression predicting PSMU scores above cut-off.

		**PSMU**	**Odds ratio**	**Adjusted odds ratio**
	***N***	***N (%)***		
**Over 35**				
Yes	5,199	245 (4.7%)	–	–
No	5,386	477 (8.9%)	1.965 (1.676–2.303)^***^	1.246 (1.032–1.505)^*^
**Gender**				
Male	5,355	361 (6.7%)	–	–
Female	5,230	361 (6.9%)	1.026 (0.882–1.193)	1.029 (0.866–1.222)
**National culture**				
Individualist	3,047	125 (4.1%)	–	–
Collectivist	7,222	527 (7.9%)	2.011 (1.649–2.452)^***^	1.537 (1.190–1.985)^***^
**Household income**				
High	4,975	312 (6.3%)	–	–
Low	5,610	410 (7.3%)	1.123 (1.012–1.371)^*^	1.082 (0.906–1.291)
**Completed college**				
No	4,717	323 (6.8%)	–	–
Yes	5,868	399 (6.8%)	1.008 (0.865–1.172)	1.123 (0.938–1.344)
**Children under 18**				
No	5,202	257 (4.9%)	–	–
Yes	5,383	465 (8.6%)	1.819 (1.554–2.129)^***^	1.535 (1.273–1.850)^***^
**Daily use-time**				
Low (<2 h)	3,027	84 (2.8.%)	–	–
High (>=2 h)	7,558	638 (8.4%)	3.230 (2.563–4.072)^***^	2.403 (1.819–3.176)^***^
**Abstention attempts**				
Yes	2,281	142 (6.2%)	–	–
No	8,304	580 (7.0%)	2.167 (1.835–2.560)^***^	1.321 (1.101–1.584)^**^
**Impact on QoL**				
Improved	7,783	467 (6.0%)	–	–
Reduced	2,802	255 (9.1%)	1.567 (1.336–1.838)^***^	1.818 (1.517–2.183)^***^

*Adjusted Odds Rartio model included all variables listed above, Ns ranged from 10,585 to 10,618 due to occasional missing data*.

*** *< 0.001*.

** *< 0.01*.

* *< 0.05*.

### Gender-Related Differences, National Culture, and Age

An exploration of gender-related differences for PSMU scores found that men (*M* = 18.08, *SD* = 5.99) and women (*M* = 17.89, *SD* = 6.01) experienced similar levels of PSMU. An independent samples *t*-test confirmed that these marginal differences in PSMU scores were not statistically significant. There were, however, minimal but statistically significant gender-related differences at the item/symptom level, with males reporting higher scores than their female counterparts for specific PSMU items relating to (2) tolerance, (4) conflict and (5) deception. Conversely, females reported higher scores than males for (6) persistence. Details of items showing gender-related differences are reported in Table 5.

**Table 5:** PSMU items with statistically significant gender-related differences.

**Item**	**Content**	***t***	***p***	***Cohen's d***
2	I need to spend increasing amount of time engaged on social media in order to enjoy it	4.209	<0.001	0.082
4	Using social media has caused problems between me and my friends and family	2.973	0.003	0.058
5	I have misled friends, family members, or others about the amount of time I spend using social media	3.277	0.001	0.064
6	I normally use social media for longer than I initially intended	−2.638	0.008	−0.051

Exploring PSMU scores and national culture (collectivist vs. individualist) revealed that participants in collectivist nations (*M* = 19.05, *SD* = 5.61) generally scored higher than their individualist counterparts (*M* = 15.39, *SD* = 6.14). An independent samples *T*-test demonstrated that these group differences were statistically significant, *t* (10,616) = −29.65, *p* < 0.001, *d* = −0.63. This finding was also replicated at the PSMU item level, with the collectivist group scoring higher than their individualist counterparts on all items, *p* < 0.001 in all instances, with effect sizes ranging from Cohen's *d* = 0.3 to 0.6. We also conducted a Pearson's bivariate correlation between the continuous values for individualism, PSMU scores and age. As hypothesized, individualism scores were negatively correlated with PSMU, *r* (10,616) = −0.275, *p* < 0.001. Similarly, PSMU scores were negatively correlated with age, *r* (10,616) = −0.332, *p* < 0.001, Finally, age was positively correlated with individualism *r* (10,616) = 0.384, *p* < 0.001. While adjusting for covariates, we undertook bivariate and multivariate logistic regression to explore these data further. This analysis also allows us to communicate an easily comprehended statistical risk model (35).

### Regression Analysis

Bivariate and multivariate logistic regressions were conducted with R (R Core Team, 2020), using generalized linear models in the base package. A binary logistic regression model was used to predict PSMU status (strong endorsement of 5 or more PSMU symptoms), computing bivariate odds ratios (OR) and multivariate adjusted odds ratios (AOR) for all predictor variables. The predictor variables were age group, gender, national culture, household income bracket, education, parent status (having children under 18 years old), social media use-time status, history of successful abstention attempts and perceived impact of social media on the quality of life (QoL). The specifics of this analysis are detailed in Table 4.

## Discussion

This work explored problematic social media use (PSMU) across 30 nations. Beyond describing international variation in prevalence, the study also sought to identify several socio-demographic and attitudinal correlates of PSMU in adult population. Such an analysis contributes to our understanding of this emerging phenomenon that has implications for psychological wellbeing (9, 10, 12, 15).

In line with previous multinational studies (27, 30), rates of PSMU varied greatly between nations. In the present study, PSMU rates varied from a high of 18.4% for India to a low of 1.7% for South Korea. Some of this variation may be explicable in terms of population age. For example, according to UN data, the median age in India, as of 2020, is 28.4, while South Korea's median age is 43.7. This difference is reflected in our sample, with the mean age for Indian respondents being 35.2, while the South Koreans were significantly older, 44.4. However, regardless of individual age, living in a nation with a relatively youthful or relatively aging population may impact the frequency or style of social media use. For example, social media use may become more broadly normalized across all age groups in a more youthful national population.

Another possible explanation for the international variability in PSMU prevalence is the differing internet penetration rates, that is, the number of internet connections per head of population. Boer et al. (30) suspected that national internet penetration rates might influence the prevalence of PSMU. However, they found no such association across 29 nations. Furthermore, South Korea has one of the highest internet penetration rates in the world. It is also worth noting that over the past decade, the South Korean government have been aggressively responsive to the issue of internet addiction, viewing it as a serious social and public health problem (36). The South Korean government was an innovator and early adopter of public policy aimed at curbing problematic internet use, with measures such as “Internet Shutdown” and “Cooling Off” aimed at limiting adolescents' nocturnal online activities (37). Perhaps the lower rates of PSMU observed for South Korea in the present study are partly due to this proactive approach to the issue.

Beyond the median age of the population and internet-related public policy, there are likely to be other within-country factors responsible for the international variability in prevalence. National culture is one such candidate variable. To test our hypothesis about the cultural differences between the nations, we examined the scores of PSMU across the 30 nations that took part in this survey. Being from a collectivist society was predictive of PSMU. Looking at the nations with the highest rates of PSMU, the top 50% are all categorized as having collectivist national cultures (28). Furthermore, four of the five nations with the lowest rates of PSMU are individualist. This pattern of findings aligns with Cheng et al. (27), where data from a large multinational meta-analysis demonstrated a similar pattern of heightened PSMU among participants from relatively collectivist nations. It might be that the values and obligations associated with collectivism such as, compliance to social norms (fitting-in) and maintaining close kinship connections (38), drives a more compulsive style of social media use in such societies, leading to higher rates of PSMU. These ideas are certainly an area for future research.

Looking at the whole sample, we could also identify six robust predictors (in the statistical sense) of PSMU across nations. Foremost among these was time spent on social media. After adjusting for all other variables in the model, those who reported spending 2 hours (intense users) or more per day on social media were more than twice as likely to score above the PSMU cut-off. Several earlier studies also report this association (6, 18, 39). However, within the present study design, it is not possible to distinguish cause from consequence. Time spent on social media may result from having developed problematic usage patterns. It might equally be a precondition or precursor to the development of PSMU among the vulnerable. The vast majority of intense users did not score above the PSMU cut-off, and previous research has even reported a positive association between intense social media use and wellbeing in specific contexts where intense use is relatively normative (30). Similarly, in the present analysis, although uncommon, some of the infrequent users also reported PSMU. What constitutes intense use is likely to vary across time and place and between individuals. For some people, 30 min of social media use may be highly ego-dystonic, likely to cause distress for themselves and significant others.

Other attitudinal and behavioral predictors included viewing social media as detrimental to one's quality of life and having never successfully attempted to abstain (detox) from social media. Like “time-spent on social media”, the inability or unwillingness to complete a period of abstinence can be viewed as a consequence of PSMU. Conversely, it might also be argued that consciously imposing periods of abstinence (prolonged breaks) on oneself offer some protection against social media use becoming problematic. In contrast, the association between perceptions of social media as negatively impacting one's quality of life is most simply explained as a possible consequence of PSMU.

We have also tested the hypothesis that PSMU is associated with younger age. Our findings showed that amongst the influential socio-demographic predictors of PSMU were younger age group, national culture and parenthood (being a parent to a minor). The association with younger age (adolescence) has been widely reported (31) and perhaps reflects generational differences in early exposure and early adoption of such emerging technologies. Although statistically significant, the age group difference in the present study had the lowest of all the adjusted odds ratios (1.2). As earlier generations increasingly adopt social media in existing users age, these age-related differences for PSMU may well fade.

The fact that parenthood was predictive of PSMU is interesting. Unfortunately, we cannot identify any previous studies that have included this as a variable, perhaps due to the pervasive focus on adolescents. In explaining the present finding, we propose that the stress associated with parenting may lead to PSMU as a form of mood-modification or escape strategy.

There is a growing body of evidence detailing lower levels of subjective wellbeing and higher stress levels among parents compared to nonparents (40). These higher stress levels are variously attributed to the time/role demands, financial burden and sleep deprivation associated with parenthood (40).

Finally, being from a collectivist society was predictive of PSMU. As discussed above, this is a finding that merits further research, and it might be that collectivist family structures and communication patterns increase the risk of PSMU.

While this study sheds new multinational cross-generational light on our understanding of PSMU, it also has several significant limitations. Firstly, the cross-sectional and correlational design means that any discussion about causation is, at best, tentative/speculative. The measure of time spent online in our study was a self-reported estimate that may potentially carry some bias. Future studies should seek opportunities to utilize objective measures of online behavior, such as using the specialized monitoring software. Using an adapted/original 8-item PSMU measure limits meaningful cross-study comparisons. Although the PSMU measure used had good face validity, and the items were drawn from well-validated existing tools, the percentage of PSMU are not directly interpretable in the context of earlier research. However, the primary aim of the present study was to look at variation between nations and identify potentially helpful correlates as a preliminary step toward informing public policy and health initiatives aimed at reducing PSMU incidence and prevalence. In our study, we did not have a variable measuring such important sociodemographic characteristic as the place of residence. Therefore, we recommend that future studies examining the cultural differences in the context of PSMU should include such a variable in their analysis to allow a thorough examination of cultural differences.

Based on our findings and the mentioned above limitations, we propose the following directions for future studies. Further studies could explore the role of culture in PSMU and perhaps examine the role of culture also in the context of other online behaviors such as internet gaming. This will allow to ascertain the observed differences and to better understand the mechanisms behind the differing risks in people from individualist vs. collectivist societies. Another area of exploration that warrants further research is related to our finding of the role of parenthood (being a parent of a minor) in PSMU. Future studies could examine what aspects of parenthood appear to drive the possible risks of PSMU associated with parenting or examine whether PSMU is a manifestation of maladaptive parenting practices or perhaps a result of other factors, such as parental psychological wellbeing.

## Data Availability Statement

The datasets presented in this article are not readily available because the ethical approval for the study requires that only anonymized data may be shared on request with verified researchers. Requests to access the datasets should be directed to Prof. Justin Thomas, Justin.Thomas@zu.ac.

## Ethics Statement

The studies involving human participants were reviewed and approved by Ithra's Internal Review Board (IRS 202171). The patients/participants provided their written informed consent to participate in this study.

## Author Contributions

JT contributed to conception of the project, analysis, and drafting of the manuscript. MV contributed to submission and review process. FA, BA, and YA contributed to the data collection and drafting of the manuscript. All authors contributed to the article and approved the submitted version.

## Funding

This study was funded by King Abdulaziz Center for World Culture (Ithra). Ithra is a non-profit organization dedicated to advancing human potential through culture and creativity.

## Conflict of Interest

The authors declare that the research was conducted in the absence of any commercial or financial relationships that could be construed as a potential conflict of interest.

## Publisher's Note

All claims expressed in this article are solely those of the authors and do not necessarily represent those of their affiliated organizations, or those of the publisher, the editors and the reviewers. Any product that may be evaluated in this article, or claim that may be made by its manufacturer, is not guaranteed or endorsed by the publisher.
